# Psychometric validation of a chinese version of COVID-19 vaccine hesitancy scale: a cross-sectional study

**DOI:** 10.1186/s12879-022-07746-z

**Published:** 2022-10-01

**Authors:** Yiman Huang, Yijin Wu, Zhenwei Dai, Weijun Xiao, Hao Wang, Mingyu Si, Wenjun Wang, Xiaofen Gu, Li Ma, Li Li, Shaokai Zhang, Chunxia Yang, Yanqin Yu, Youlin Qiao, Xiaoyou Su

**Affiliations:** 1 School of Population Medicine and Public Health, Chinese Academy of Medical Sciences and Peking Union Medical College, Beijing, China; 2 School of Nursing, Jining Medical University, Jining, Shandong China; 3 Affiliated Tumor Hospital, Xinjiang Medical University, Urumqi, China; 4 School of Public Health, Dalian Medical University, Dalian, China; 5 Department of Clinical Research, The First Affiliated Hospital, Jinan University, Guangzhou, China; 6 Henan Cancer Hospital, Affiliated Cancer Hospital of Zhengzhou University, Zhengzhou, China; 7 Department of Epidemiology and Biostatistics, School of Public Health and West China Fourth Hospital, Sichuan University, Chengdu, Sichuan China; 8 School of Public Health, Department of Clinical Research, Baotou Medical College, Baotou, China

**Keywords:** COVID-19, Vaccine hesitancy, Scale, Validation

## Abstract

**Background:**

COVID-19 vaccines have been administered in many countries; however, a sufficient vaccine coverage rate is not guaranteed due to vaccine hesitancy. To improve the uptake rate of COVID-19 vaccine, it is essential to evaluate the rate of vaccine hesitancy and explore relevant factors in different populations. An urgent need is to measure COVID-19 vaccine hesitancy among different population groups, hence a validated scale for measuring COVID-19 vaccine hesitancy is necessary. The present study aims to validate the COVID-19 vaccine hesitancy scale among different populations in China and to provide a scale measuring COVID-19 vaccine hesitancy with satisfactory reliability and validity.

**Methods:**

Self-reported survey data were collected from different populations in China from January to March 2021. Based on the Parent Attitudes about Childhood Vaccines scale, 15 items were adapted to evaluate the COVID-19 vaccine hesitancy. Exploratory and confirmatory factor analysis were utilized to identify internal constructs of the COVID-19 vaccine hesitancy scale among two randomly split subsets of the overall sample. Reliability was analyzed with the internal consistency, composite reliability, and the test–retest reliability, and validity was analyzed with the criterion validity, convergent validity, and discriminant validity.

**Results:**

A total of 4227 participants completed the survey, with 62.8% being medical workers, 17.8% being students, 10.3% being general population, and 9.1% being public health professionals. The exploratory factor analysis revealed a three-factor structure that explain 50.371% of the total variance. The confirmatory factor analysis showed that models consisting of three dimensions constructed in different populations had good or acceptable fit (CFI ranged from 0.902 to 0.929, RMSEA ranged from 0.061 to 0.069, and TLI ranged from 0.874 to 0.912). The Cronbach’s α for the total scale and the three subscales was 0.756, 0.813, 0.774 and 0.705, respectively. Moreover, the COVID-19 vaccine hesitancy scale had adequate test–retest reliability, criterion validity, convergent validity, and discriminant validity.

**Conclusions:**

The COVID-19 vaccine hesitancy scale is a valid and reliable scale for identifying COVID-19 vaccine hesitancy among different population groups in China. Given the serious consequences of COVID-19 vaccine hesitancy, future studies should validate it across regions and time to better understand the application of the COVID-19 vaccine hesitancy scale.

**Supplementary Information:**

The online version contains supplementary material available at 10.1186/s12879-022-07746-z.

## Background

Coronavirus disease 2019 (COVID-19) has been wreaking havoc on the world, taking a heavy toll on human lives and economic activities. Countries in different regions are facing the challenge of containing its spread. To curb the COVID-19 pandemic, COVID-19 vaccines have been successfully developed and are being administered in many countries. The COVAX Facility, led by WHO, is designed to guarantee rapid, fair and equitable access to COVID-19 vaccines for every country in the world to achieve WHO’s vaccine equity target [1]. However, it’s not vaccines that will stop the pandemic, it’s vaccination. Vaccination is considered to be one of the greatest achievements of public health and has contributed to the decline in mortality and morbidity of various infectious diseases. COVID-19 vaccine hesitancy may be a barrier to sustained public acceptance of vaccination and could lead to an insufficient vaccine coverage rate to achieve herd immunity [2].

Vaccine hesitancy refers to a refusal or delay in acceptance of vaccination despite the availability of vaccination services. It is complex and context-specific, varying across time, place, and vaccines [3]. A certain amount of the population has been hesitant or unsure about vaccination since the first vaccine was made available, and those who are hesitant about vaccines may have varied levels of hesitancy about specific vaccines or vaccination in general [4, 5]. Vaccine hesitancy has emerged as a major public health crisis in recent decades, and World Health Organization (WHO) has listed it as one of the ten leading threats to global health in 2019 [6]. Indeed, vaccine hesitancy has a negative impact on maintaining herd immunity, preventing outbreaks of vaccine-preventable diseases, and ensuring the vaccination of novel vaccines [7].

Regarding the factors related to vaccine hesitancy, WHO identified complacency, inconvenience in access, and a lack of confidence as the driving factors [6]. The complacency refers to the belief that perceived risks of vaccine-preventable diseases are low and that vaccination is not a necessary preventive action; the convenience refers to vaccine availability and accessibility; and the confidence refers to the trust in the effectiveness and safety of vaccines, the delivery system, and the motivations of vaccination policymakers [8, 9]. Previous studies demonstrated that complacency, convenience, and confidence were equally significant factors influencing COVID-19 vaccine hesitancy [10, 11]. In addition, COVID-19 vaccine hesitancy was also depended on a variety of factors, such as concerns about side effects, perceptions of the benefits, perceptions of vaccination risks or harms, trust in authorities, vaccination knowledge, and their own vaccination history [12–17].

To measure COVID-19 vaccine hesitancy, most studies utilized one-item question: “Would the respondents be willing to vaccinate against COVID-19 if COVID-19 vaccine is available?” [18–21]. Several studies used the Vaccine Hesitancy Scale (VHS) or modified VHS, a 10-item scale developed to measure parental vaccine hesitancy [22, 23], and to measure adult’s COVID-19 vaccine hesitancy [24–26]. The Oxford COVID-19 vaccine hesitancy scale was a newly developed scale which comprises 7 items and focused mostly on the link between hesitancy and vaccine conspiracy beliefs to measure COVID-19 vaccine hesitancy among adults [27–29]. The COVID-19 vaccine attitudes and beliefs scale was proposed to measure adults’ hesitancy, consisting of 20 items in four dimensions: general COVID-19 vaccination beliefs and attitudes, COVID-19 vaccination adverse effects, perceived knowledge sufficiency, and return to “normal” life [30, 31]. However, there are no data on adapted or validated scales capable of assessing vaccine hesitancy among different populations, and a valid scale to identify COVID-19 vaccine hesitancy that could perform reliably across diverse populations would be especially valuable.

At present, WHO points out that COVID-19 transmission remains very high, vaccination coverage remains very low in too many countries, and certain numbers of healthcare workers and others at high risk are still unvaccinated, with the risk of new variants emerging [32]. Measuring different populations’ hesitancy levels to receive the COVID-19 vaccine is essential to better understand the reasons for it and further increase the vaccination rate. The aim of the current study was to adapt and validate a COVID-19 vaccine hesitancy scale based on the Parent Attitudes about Childhood Vaccines (PACV) scale to measure COVID-19 vaccine hesitancy and then validated it among 4 different population groups in China. The PACV was originally developed by adapting items from a previous Health Beliefs Survey in 2011, which included an 18-item survey encompassing the domains: immunization behaviors, beliefs about vaccine safety and effectiveness, attitudes, and trust [33]. Then Larson et al. adapted and validated a series of questions for greater global relevance on this basis in 2015, the results of this process was a 15-item scale, containing 15 items in 3 dimensions: general attitudes, safety and efficacy, and behavior. And PACV has been utilized or validated in different countries, such as China, Italy, Malaysia, and Switzerland [34–38]. Potentially, a COVID-19 vaccine hesitancy scale with satisfactory reliability and validity could provide timely feedback to health authorities and policy makers by assessing COVID-19 vaccine hesitancy in order to establish appropriate strategies to address vaccine hesitancy during the COVID-19 pandemic.

## Methods

### Survey design and sampling

#### Sample size

Exploratory factor analysis requires a sample size 10 times larger than the number of items [39]. Given the number of items in the scale was 15, the sample size was planned 150 at least for exploratory factor analysis among each studied population. The sample size for confirmatory factor analysis was planned 192 at least among each population group, with the set of $\alpha$= 0.05, $\beta$ = 0.20, df = 87, RMSEA = 0.05 in the null hypothesis, and RMSEA = 0.01 in the alternative hypothesis [40, 41].

#### Sampling strategy

Firstly, the 31 provinces/municipalities/autonomous regions of China are divided into seven geographical divisions, namely, East China, South China, Central China, North China, Northwest China, Southwest China, and Northeast China. Second, in each of the 7 geographic divisions, one representative city is selected, which are Dalian, Jinan, Shenzhen, Sichuan, Xinjiang, Henan, and Inner Mongolia in China. Finally, snowball sampling was used to recruit the potential study participants. Investigators from the seven cooperative institutions were initially invited, and they distributed the questionnaire to those who met the inclusion criteria.

#### Participants recruitment

The cross-sectional survey was conducted in seven cities by generating an electronic questionnaire on a survey platform named Wenjuanxing. Then, investigators shared the link of questionnaire on WeChat among the potential participants. The participants included four different population groups: students, public health professionals, medical workers, and the general population. The medical workers were recruited from hospital departments such as respiratory and critical care medicine, general surgery, and nephrology department, while hospital administrators were excluded from medical workers group in our study. The public health professionals were recruited from local CDCs in China, mostly from the communicable disease control and prevention department, immunization program department, and preventive health department. The student group was recruited from students of local universities. The retest sample representing four different populations in seven cities was repeatedly surveyed over a four-week period [42]. People who were 18 years old or above and could read, understand and complete an online questionnaire were eligible to participate. Those who were younger than 18 years old, had difficulty using a cell phone or computer, or had cognitive impairments were excluded. To ensure the quality of the online survey, study team members received training on standard procedures for data collection and inclusion.

This study has been approved by the Ethical Review Committee of Chinese Center for Disease Control and Prevention on December 4th, 2020 (approval number: 202020). An electronic informed consent was provided before the start of the questionnaire survey. Upon completion of the informed consent, the study participants filled in the online questionnaire.

### Measurements

#### Sociodemographic information

Sociodemographic variables included age, gender (male or female), ethnicity (Han ethnicity or other), residence place (urban or rural), marital status (single, married, or others), education level (below high school or college and above), and household income (during past 1 year).

#### The COVID-19 vaccine hesitancy scale (CVHS)–the modified scale based on PACV

We used 15 items to identify the COVID-19 vaccine hesitancy, these items were adapted from the Parent Attitudes About Childhood Vaccines (PACV) scale, which was originally designed to measure the parents’ attitude towards childhood vaccines by the WHO Strategic Advisory Group on Experts (SAGE). The revised 15-item scale was named the COVID-19 vaccine hesitancy scale (CVHS). Based on the PACV, the revised 15-item scale was composed of the three domains: “Safety and Efficacy” with 4 items, “General Attitudes” with 9 items, and “Behavior” with 2 items [22, 33, 38]. Responses of scale items was divided into 3 categories: hesitant responses, “not sure or don’t know”, and non-hesitant responses. Additional file 1: Appendix A contains the specific items and scoring rules of this scale. The raw total score was calculated by summing up each item’s score, ranging from 0 to 30. Simple linear transformation was used to convert this raw score to a 0–100 scale, accounting for missing values. COVID-19 vaccine hesitancy was indicated by a score higher than or equal to 50 [33].

#### The revised flu vaccine hesitancy scale for measurement of COVID-19 vaccine hesitancy

On the basis of the flu vaccine hesitancy scale, we replaced “flu vaccine” in the items with “COVID-19 vaccine” to form the revised flu vaccine hesitancy scale. The flu vaccine hesitancy scale is comprised of 6 items and 3 dimensions (complacency, confidence and convenience) [43]. In this study, complacency was measured by perceived necessity and importance of the vaccine; confidence was measured by perceived vaccine safety and effectiveness; convenience was measured by perceived convenience and affordability of the vaccine. Participants rated each item on a 5-point Likert scale (1 = strongly disagree; 5 = strongly agree). The items were as follows: (1) necessity: “Thinking specifically about the COVID-19 vaccine, do you think the COVID-19 vaccine is necessary?”, (2) importance: “Thinking specifically about the COVID-19 vaccine, do you think the COVID-19 vaccine is important?”, (3) safety: “Thinking specifically about the COVID-19 vaccine, do you think the COVID-19 vaccine is safe?”, (4) effectiveness: “Thinking specifically about the COVID-19 vaccine, do you think the COVID-19 vaccine is effective?”, (5) convenience: “Thinking specifically about the COVID-19 vaccine, do you think the COVID-19 vaccine is convenient?”, (6) affordability: “Thinking specifically about the COVID-19 vaccine, do you think the COVID-19 vaccine is affordable?”. In the current study, the revised scale had satisfactory reliability (Cronbach’s α = 0.942), and the Cronbach’s α values for the three dimensions were 0.930, 0.925 and 0.820, respectively.

#### The vaccine confidence scale

The vaccine confidence scale consisted of 8 items on an 11-point response scale ranging from 0 (“strongly disagree”) to 10 (“strongly agree”), assessing three domains of the benefits of vaccination ( “Benefits”), the harms of vaccination (“Harms”), and trust in healthcare providers (“Trust”) [44]. Using the Vaccination Confidence Scale, we reverse-coded the negative attitudes in the Harms factor and calculated mean scores for each participant by averaging responses for all 8 items. The resulting scores had a possible range of 0 to 10 with higher scores indicating more positive attitudes about vaccination [45]. In this study, it has good reliability (Cronbach’s α = 0.774), and the Cronbach’s α values for the three dimensions were 0.592, 0.857 and 0.803, respectively.

### Translation of the COVID-19 vaccine hesitancy scale and pilot testing

First, two translators independently translated the scale from English to Chinese. These translators were bilingual, with professional competence in English and native proficiency in Chinese. Then, the two Chinese versions were combined into one Chinese version, and experts from seven partner institutions were invited to review the Chinese version and make partial changes to the wording of the questions in it, so that the scale could be accurately translated into the Chinese version. And a pilot test of the preliminary version was conducted in 7 cities among different populations. These participants were invited to comment on any questions that they found difficult to understand, difficult to answer, disturbing, confusing, or offensive. No major problems were found in the pilot test and only minor modifications were made to finalize the Chinese version.

### Data analysis

The sample was randomly divided into two parts for performing exploratory factor analysis and confirmatory factor analysis respectively, and to identify factors and further test the factor structure of the scale. A random number generator was employed to divide the sample into two groups with a 50/50 split to ensure its equal distribution. The final sample size of the exploratory sample (sample 1) was 2123 and the confirmatory sample (sample 2) was 2104. Descriptive statistics such as mean (standard deviation) and frequency were used to elaborate the sociodemographic characteristics of different population groups. The level of significance was set at *P* < 0.05. AMOS Version 24.0 and SPSS Version 24.0 were used to perform the analysis.

### Exploratory factor analysis

The KMO (Kaiser–Meyer–Olkin) measurement of sampling adequacy was used to test whether our data were suitable for EFA [46]. Exploratory factor analysis for the scale was conducted in sample1 using principal components analysis with varimax-based rotation to account for possible correlations among factors. Items which had a factor loading of more than 0.40 and did not load on multiple factors were considered part of a factor [47].

### Confirmatory factor analysis

Confirmatory factor analysis was then conducted in sample 2 among four different population groups (students, public health professionals, medical workers, and the general public) in order to demonstrate that the factor structure validates across an independent sample of different populations. Model fit was evaluated by a few goodness of fit indices, including the root mean square error of approximation (RMSEA), the comparative fit index (CFI), and the Tucker Lewis index (TLI) [48–50]. RMSEA values close to 0.06, or below, were regarded as good fit, 0.07 to 0.08 as moderate fit, 0.08 to 0.10 as marginal fit, and > 0.10 as poor fit [51]. For the CFI and TLI, values close to 0.95 or above were regarded as good fit, values close to 0.90 and 0.95 as acceptable fit, and values approaching 0 as poor fit [52, 53].

### Reliability

For the evaluation of the reliability of the scales, Cronbach’s alphas were calculated across the overall scale and each subscale to determine the internal consistency (Cronbach’s α ≥ 0.70 was considered satisfied) [54]. And Composite Reliability (CR) for each factor was also calculated to evaluate the reliability (CR $>$ 0.70 was considered satisfied).

### Convergent and discriminant validity

Convergent validity was assessed by Average Variance Extracted (AVE) and CR, and convergent validity was considered high if AVE was greater than 0.50, CR was greater than 0.70, and CR was greater than AVE. Discriminant validity was considered satisfactory if the correlation between the factor scores was significant and the correlation coefficient was less than the square root of the corresponding AVE [55, 56].

### Criterion validity

Criterion validity was examined through bivariate Pearson correlation analysis between the CVHS and the revised flu vaccine hesitancy scale, and correlations between the CVHS and the vaccine confidence scale. Significant correlations between the CVHS and the revised flu vaccine hesitancy scale and the vaccine confidence scale indicated adequate construct validity.

### Test–retest reliability

Test–retest reliability was probed by the intraclass correlation coefficients (ICCs) of the self-reported scores of retest samples that were repeatedly surveyed over a 4-week period in 4 different populations from 7 cities. ICCs above 0.40 were acceptable; ICCs above 0.60 or greater indicated satisfactory stability; and ICCs greater than 0.80 were excellent [57].

## Results

### Sociodemographic information

The sociodemographic characteristics of samples were summarized in Table 1. A total of 4289 respondents (response rate 95.37%) completed the online questionnaire, and 62 questionnaires were excluded due to age limitations. Among them, 62.8% were medical workers, 17.8% were students, 10.3% were general population, and 9.1% were public health professionals. The mean age of all participants was 33.02 years old. A total of 2818 (66.7%) respondents were female and 33.3% of them were male. 89.1% of them were of Han ethnicity. The majority of participants (85.6%) lived in urban areas. 56.9% of them were married and 41.3% were single. The education level of most of them (93.8%) were college or above. For the household income during the past 1 year, 43.4% of them were in the range of 50,000–100,000 Yuan per year, while 33.8% of them were in the range of 110,000–350,000 Yuan.

**Table 1:** Sociodemographic characteristics of participants by different population groups

Variables	Medical worker (n = 2656)	Students (n = 753)	General population (n = 434)	Public health professionals (n = 384)	Total (n = 4227)
Age (years) ($mean\pm SD)$	35.89 $\pm$ 9.33	22.47 $\pm$ 3.13	29.73 $\pm$ 7.78	37.52 $\pm$ 9.03	33.02 $\pm$ 9.90
Gender, n (%)					
Male	732 (27.6)	334 (44.4)	193 (44.5)	150 (39.1)	1409 (33.3)
Female	1924 (72.4)	419 (55.6)	241 (55.5)	234 (60.9)	2818 (66.7)
Ethnicity, n (%)					
Han	2339 (88.1)	654 (86.9)	415 (95.6)	357 (93.0)	3765 (89.1)
Other	317 (11.9)	99 (13.1)	19 (4.4)	27 (7.0)	462 (10.9)
Residence place, n (%)					
Urban	2408 (90.7)	496 (65.9)	355 (81.8)	361 (94.0)	3620 (85.6)
Rural	248 (9.3)	257(34.1)	79 (18.2)	23 (6.0)	607 (14.4)
Marital status, n (%)					
Single	667 (25.1)	721 (95.8)	263 (60.6)	93 (24.2)	1744 (41.3)
Married	1927 (72.6)	27 (3.6)	165 (38.0)	285 (74.2)	2404 (56.9)
Others	62 (2.3)	5 (0.7)	6 (1.4)	6 (1.6)	79 (1.9)
Education level, n (%)					
≤ High school	160 (6.0)	25 (3.3)	53 (12.2)	22 (5.7)	260 (6.2)
College or above	2496 (94.0)	728 (96.7)	381 (87.8)	362 (94.3)	3967 (93.8)
Household income (past 1 year), n (%)					
≤ 40,000 Yuan	431 (16.2)	256 (34.0)	84 (19.4)	43 (11.2)	814 (19.3)
50,000–100,000 Yuan	1233 (46.4)	285 (37.8)	185 (42.6)	132 (34.4)	1835 (43.4)
110,000–350,000 Yuan	920 (34.6)	178 (23.6)	137 (31.6)	193 (50.3)	1428 (33.8)
> 350,000 Yuan	72 (2.7)	34 (4.5)	28 (6.5)	16 (4.2)	150 (3.5)

### Exploratory factor analysis

The value of KMO measure of sampling adequacy was 0.755 and Bartlett’s test of Sphericity was significant (*P* < 0.001). The total variance of the 15 items explained by the 3 factors was 50.371%. Table 2 showed the factor loadings of the 15 items to the three factors extracted. The factor loadings of the 15 items were ranging from 0.294 to 0.880, and excepted the 6th and 12th item, all other items loaded on the expected factors as the original dimensions (> 0.40). The 6th and the 12th item had relatively weak loadings on the “General Attitudes” factor, they were hence classified as the “Safety and efficacy” factor.

**Table 2:** Exploratory factor analysis with loadings of 15 items (n = 2123)

	Subscales		
	Behavior	Safety and efficacy	General Attitudes
T1	**0.880**	0.042	0.048
T2	**0.876**	0.040	0.108
T3	0.239	0.049	**0.703**
T4	0.084	0.211	**0.421**
T5	0.231	− 0.335	**0.421**
T6	− 0.047	**0.294**	0.181
T7	− 0.077	**0.451**	− 0.042
T8	0.108	**0.844**	0.010
T9	0.146	**0.870**	0.073
T10	0.124	**0.795**	0.128
T11	0.064	0.226	**0.647**
T12	0.342	**0.394**	0.377
T13	− 0.042	− 0.006	**0.711**
T14	− 0.123	− 0.017	**0.660**
T15	0.161	0.027	**0.702**

Value in bold type means that its corresponding item is loaded on its corresponding subscale

### Confirmatory factor analysis

The confirmatory factor analyses based on the original structure of “Safety and efficacy” dimension (4 items), “General Attitudes” dimension (9 items), and “Behavior” dimension (2 items) were performed in sample 2 among four different population groups. Initially, the results showed that the CFA analysis conducted among four different population groups and the whole sample 2 did not meet the criteria of RMSEA values close to 0.06 and CFI and TLI values greater than or equal to 0.90. Based on the results of EFA, we constructed new models that “Safety and efficacy” dimension included 6 items (added the 6th and 12th item), “General Attitudes” dimension included 7 items (excluded the 6th and 12th item), “Behavior” dimension included 2 items. However, the results of the new models based on the results of EFA still did not meet the above criteria and were worse compared with the original structure. The results of modification indices of the CFA model based on the original structure indicated that freeing error terms to covary could substantially improve model fit. After modification was made to the CFA model based on the original structure, RMSEA indicated the modification models were good fit, CFI and TLI indicated the modification models were acceptable fit (Table 3).

**Table 3:** Model fit indices of confirmatory factor analysis models

	Original dimensions				New dimensions based on EFA results				Original dimensions (modificated)			
	CMIN/DF	CFI	TLI	RMSEA	CMIN/DF	CFI	TLI	RMSEA	CMIN/DF	CFI	TLI	RMSEA
Model 1	17.527	0.845	0.813	0.089	18.941	0.832	0.797	0.092	11.052	0.909	0.886	0.069
Model 2	12.494	0.828	0.792	0.093	13.012	0.820	0.783	0.095	6.390	0.922	0.903	0.064
Model 3	4.030	0.812	0.773	0.092	4.381	0.790	0.747	0.098	2.683	0.902	0.874	0.069
Model 4	2.840	0.866	0.838	0.089	2.973	0.856	0.826	0.092	2.033	0.927	0.909	0.067
Model 5	2.169	0.879	0.854	0.079	2.407	0.854	0.824	0.086	1.702	0.929	0.912	0.061

Model 1: Confirmatory factor analysis among all confirmatory samples; Model 2: Confirmatory factor analysis among medical workers; Model 3: Confirmatory factor analysis among students; Model 4: Confirmatory factor analysis among general population; Model 5: Confirmatory factor analysis among public health professionals

**Fig. 1:**
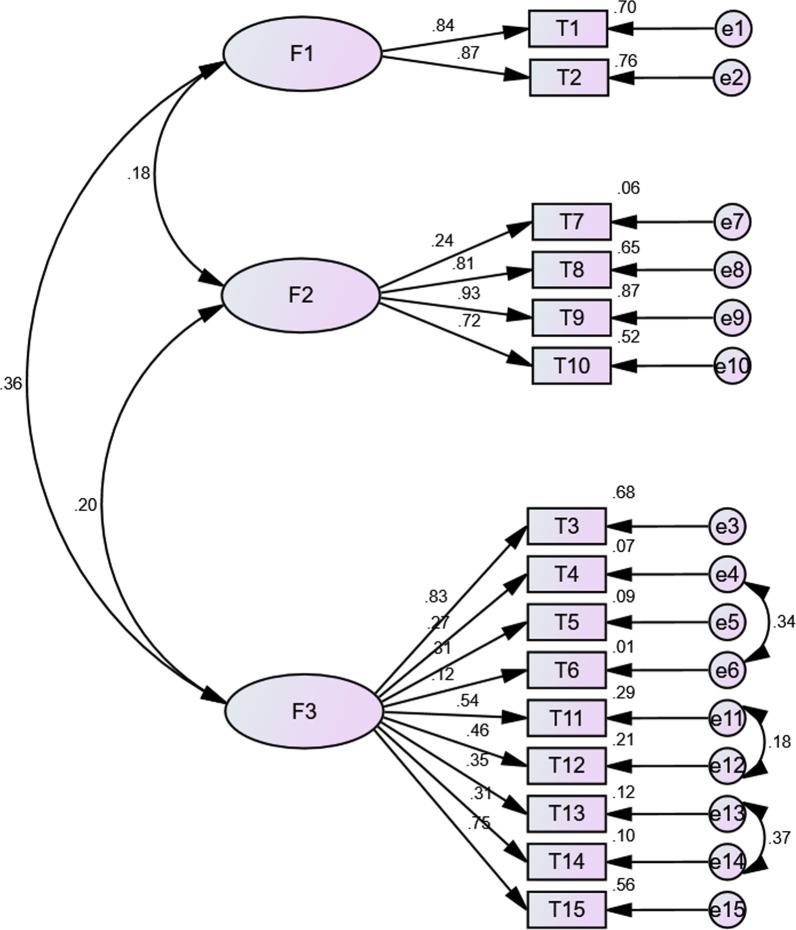
Confirmatory factor analysis among all confirmatory samples (n = 2104, standardized estimates). (F1: Behavior; F2: Safety and Efficacy; F3: General Attitudes)

**Fig. 2:**
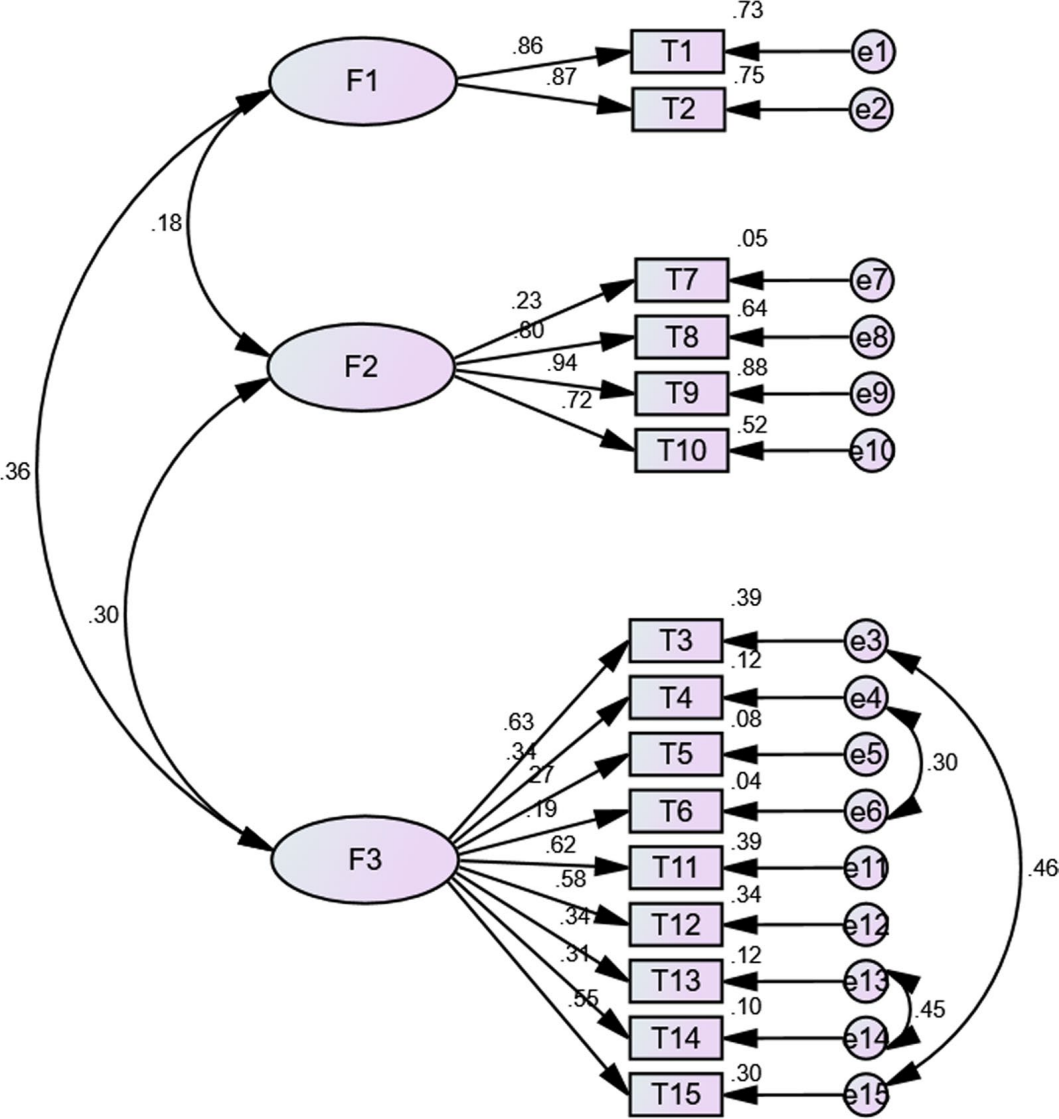
Confirmatory factor analysis among medical workers (n = 1325, standardized estimates). (F1: Behavior; F2: Safety and Efficacy; F3: General Attitudes)

**Fig. 3:**
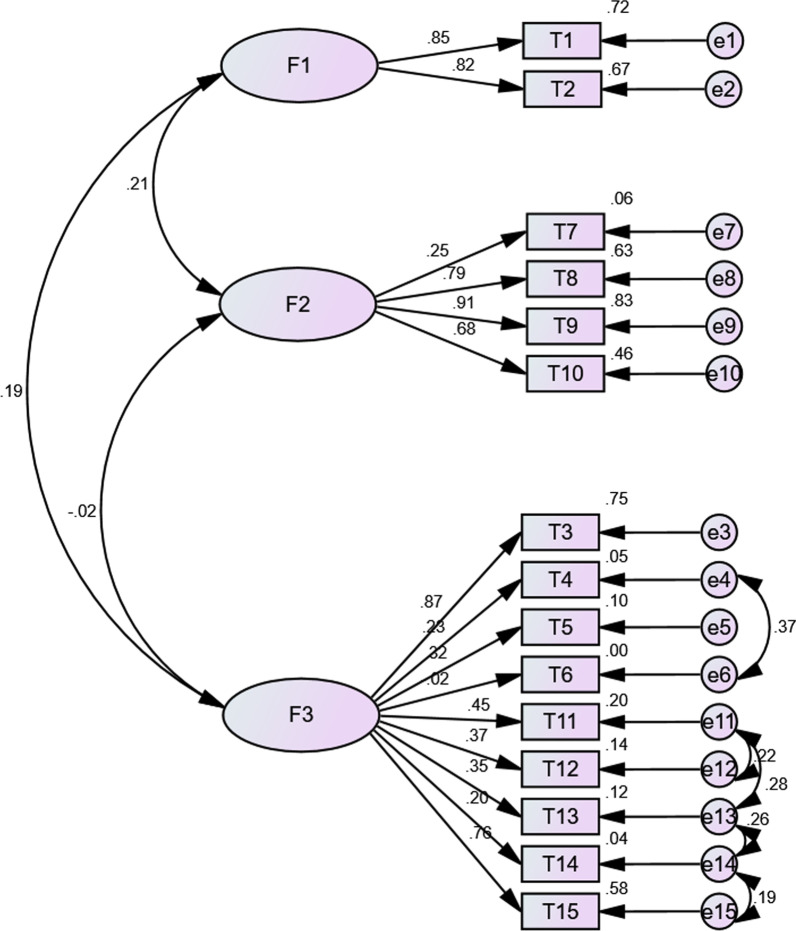
Confirmatory factor analysis among students (n = 356, standardized estimates). (F1: Behavior; F2: Safety and Efficacy; F3: General Attitudes)

**Fig. 4:**
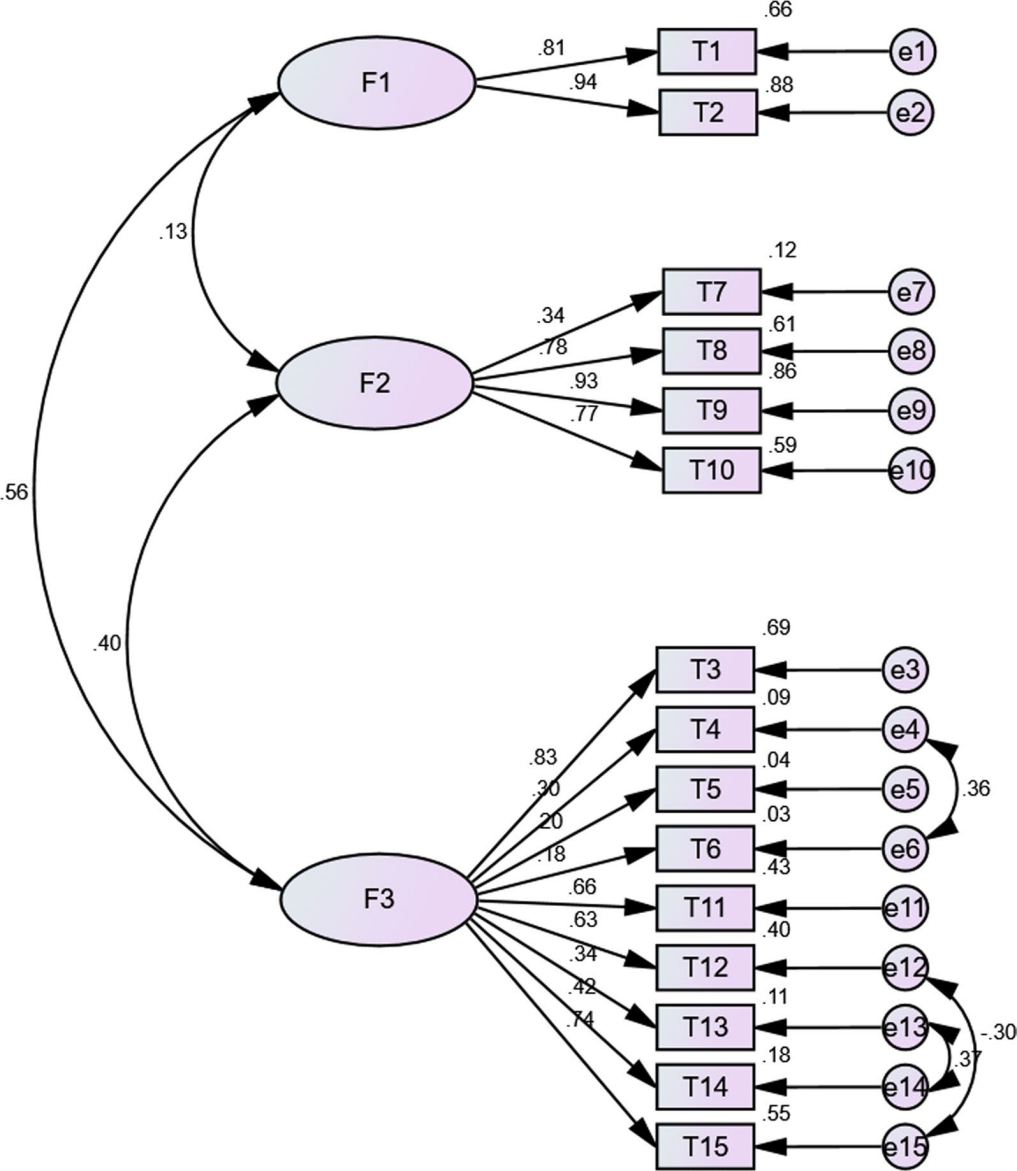
Confirmatory factor analysis among general population (n = 234, standardized estimates). (F1: Behavior; F2: Safety and Efficacy; F3: General Attitudes)

**Fig. 5:**
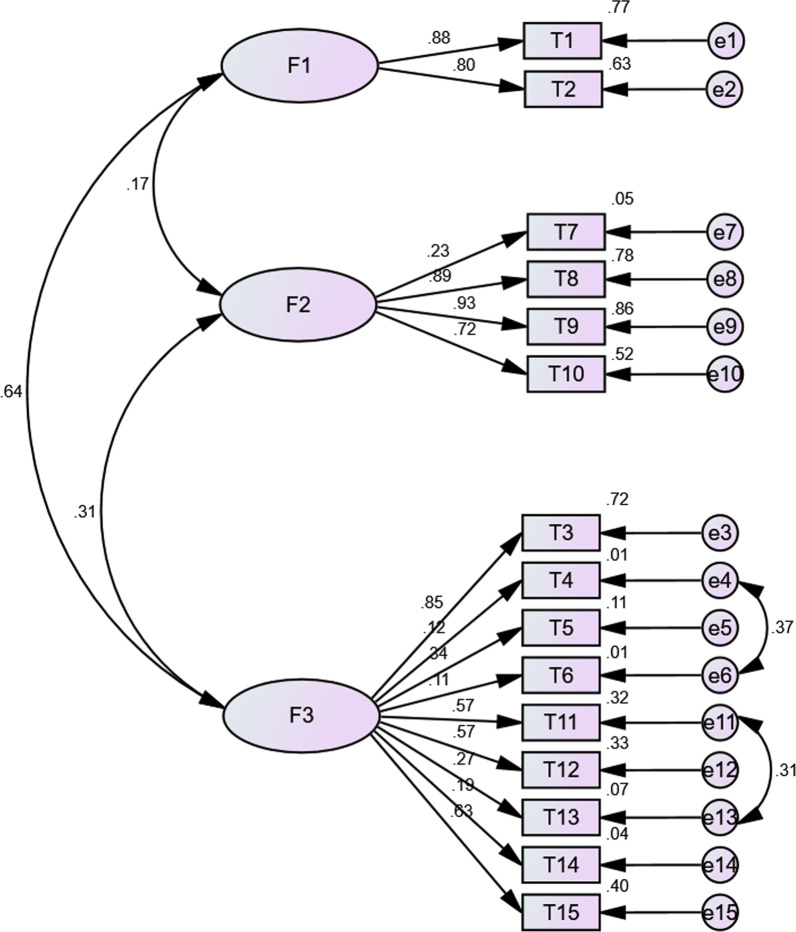
Confirmatory factor analysis among public health professionals (n = 189, standardized estimates). (F1: Behavior; F2: Safety and Efficacy; F3: General Attitudes)

Figure 1 depicted the results of the modification model built in overall sample 2, including the three dimensions and its normalization. Model 1 built in overall sample 2 fit the data well, with the value of RMSEA of 0.069, the CFI of 0.909, and the TLI of 0.886. Besides, the fit indices of the modification model based on medical workers (RMSEA = 0.064, CFI = 0.922, TLI = 0.903), students (RMSEA = 0.069, CFI = 0.902, TLI = 0.874), the general population (RMSEA = 0.067, CFI = 0.927, TLI = 0.909) and public health professionals (RMSEA = 0.061, CFI = 0.929, TLI = 0.912) samples indicated good model fit, as shown in Figs. 2, 3, 4 and 5, respectively.

### Reliability

The results of the internal consistency analysis for the overall scale demonstrated satisfactory internal consistency, with Cronbach’s α of 0.756. And the values of Cronbach’s α of the three sub-scales (“Behavior”, “Safety and Efficacy”, “General Attitudes”) were 0.813, 0.774, and 0.705, respectively. All five models had CRs greater than 0.70 for all three dimensions, with the exception of the “general attitude” dimension of the models conducted among health workers, students, public health professionals, and all confirmatory samples, which had CRs below 0.70.

### Convergent and discriminant validity

Table 4 showed the results of convergent and discriminant validity. For the convergent validity, the AVE values for the “Behavior” and “Safety and Efficacy” dimensions of the 5 models conducted in the medical workers, students, general population, public health professionals, and all confirmatory samples were greater than 0.50, except for the AVE value of 0.494 for the “Safety and efficacy” dimension of the model built in the student sample, while regarding the “General Attitudes” dimension, its AVE was lower than 0.50 in all 5 models. The CR values for the three dimensions of the five models were greater than 0.70, except for the CR of the “General Attitudes” dimension of the models conducted in medical workers, students, public health professionals and all confirmatory samples, which were below 0.70. In addition, the CR values in all factors among the 5 models were all greater than AVE. For the discriminant validity, the correlation coefficients of any two dimensions in the five populations are less than the square root of the corresponding AVE.

**Table 4:** The results of convergent and discriminant validity

	Pearson correlation coefficient			AVE	CR
	F1	F2	F3		
Medical workers					
F1	**0.865**			0.748	0.860
F2	0.155^**^	**0.724**		0.524	0.792
F3	0.277^**^	0.233^**^	**0.454**	0.206	0.673
Students					
F1	**0.835**			0.697	0.822
F2	0.198^**^	**0.703**		0.494	0.774
F3	0.211^**^	0.091	**0.470**	0.221	0.645
General population					
F1	**0.883**			0.780	0.863
F2	0.155^*^	**0.738**		0.545	0.814
F3	0.434^**^	0.334^**^	**0.529**	0.280	0.741
Public health professionals					
F1	**0.841**			0.707	0.828
F2	0.176^*^	**0.746**		0.557	0.812
F3	0.431^**^	0.319^**^	**0.473**	0.224	0.656
All confirmatory samples					
F1	**0.855**			0.731	0.845
F2	0.166^**^	**0.724**		0.524	0.793
F3	0.300^**^	0.244^**^	**0.490**	0.240	0.694

F1: Behavior; F2: Safety and Efficacy; F3: General Attitudes

*P < 0.05; **P < 0.01

On the diagonal, we inserted the square roots of every AVE value to compare it with the other correlation coefficients

Values in bold type mean that the square root of the AVE value for each subscale is higher than the correlation coefficients with the other subscales

### Criterion validity

Table 5 showed the results of bivariate Pearson analysis between the COVID-19 vaccine hesitancy scale and the revised flu vaccine hesitancy and the vaccine confidence scale. For the revised flu vaccine hesitancy scale, the results showed that the three subscales of the COVID-19 vaccine hesitancy scale (the “Behavior”, “Safety and Efficacy”, and “General Attitudes”), and the total score of the COVID-19 vaccine hesitancy scale were positively correlated with the “Complacency” subscale, and negatively correlated with the “Confidence” and “Convenience” subscales, as well as the total scores of the revised flu vaccine hesitancy scale. For the vaccine confidence scale, the COVID-19 vaccine hesitancy scale total scores and its three subscales were found to have a significantly negative correlation with the vaccine confidence scale total score and scores of its three subscales (“Harms”, “Benefits”, “Trust”).

**Table 5:** Criterion validity results of the COVID-19 vaccine hesitancy scale (bivariate Pearson correlation analysis)

	The revised flu vaccine hesitancy scale				The vaccine confidence scale			
	Complacency	Confidence	Convenience	Total score	Harms	Benefits	Trust	Total score
Behavior	0.153	− 0.167	− 0.094	− 0.086	− 0.156	− 0.153	− 0.124	− 0.203
Safety and Efficacy	0.050	− 0.160	− 0.076	− 0.152	− 0.293	− 0.092	− 0.103	− 0.209
General Attitudes	0.571	− 0.558	− 0.492	− 0.382	− 0.304	− 0.555	− 0.483	− 0.637
Total score	0.433	− 0.481	− 0.379	− 0.343	− 0.379	− 0.442	− 0.394	− 0.566

All *P* < 0.05

### Test–retest reliability

The ICCs of the overall COVID-19 vaccine hesitancy scale for a 4-week interval among 45 participants was 0.773 (*P* < 0.001), indicating satisfactory stability. The three subscales also yielded test–retest reliabilities of 0.878 (*P* < 0.001), 0.530 (*P* < 0.05), 0.746 (*P* < 0.001), respectively.

## Discussion

In the current study, the COVID-19 vaccine hesitancy scale was modified based on the items of Parent Attitudes about Childhood Vaccines scale to evaluate people’s hesitancy about the COVID-19 vaccine among different populations, thus satisfying the need to validate an appropriately structured and specific instrument during the COVID-19 pandemic. Based on the results of EFA and CFA, the original structure reflected well on its conceptual framework. In addition, Cronbach’s alpha values for the total scale and the three subscales showed satisfactory internal consistency and also confirmed that the 15 items based on the original dimensions could consistently measure COVID-19 vaccine hesitancy. Therefore, the scale based on the initial dimensions could provide a consistent and reliable assessment of the COVID-19 vaccine hesitancy among various population in China. Convergent and discriminant validity was assessed by the AVE and CR, indicating that the scale has good stability in reflecting the measured outcomes. Criterion validity was demonstrated by the significant correlations between the CVHS overall scale and its subscales and the revised flu vaccine hesitancy scale and its three subscales (complacency, confidence and convenience), as well as its significant correlation with the vaccine confidence scale and its subscales (harms, benefits and trust). The results of the current study demonstrated the validity and reliability of the COVID-19 vaccine hesitancy scale as a reliable multifactor instrument to measure COVID-19 vaccine hesitancy.

Results of the criterion validity indicated that complacency about the vaccination were positively associated with COVID-19 vaccine hesitancy. Complacent individuals who feel that the risks are minimal are less likely to take preventive measures. Previous studies also indicated that complacency is negatively related to the adoption of preventive behavior [10, 58]. The public might become complacent after the vaccine becomes available, since they believe that COVID-19 is preventable and the consequences of infection are not severe [59]. This phenomenon may due to their limited knowledge about the risk of getting COVID-19 and the efficacy of COVID-19 vaccine, thus giving them a misperception of the disease and of the vaccine [60]. Alternatively, they may think that the surrounding people’s vaccination is sufficient to prevent transmission and protect themselves from COVID-19 infection, and so they believe they are at low risk of contracting COVID-19. Besides, people who believed in their own immunity were hesitant to get vaccinated, due to their low perception of the risk of getting the COVID-19 [61]. In addition, the large number and diversity of effective preventive measures, such as wearing masks, provide an alternative to prevent COVID-19, potentially weakening the intent or perceived necessity for vaccination for some people [62]. To reduce the complacency on vaccination, proactive public health campaigns and communications, together with appropriate social media engagement, would be efficacious solutions. The healthcare system and relevant authorities should provide more solid knowledge about COVID-19 and COVID-19 vaccines, as well as disseminate more transparent and accurate information to increase the public’s awareness of the infection risk, necessity, and the significance of vaccination as well.

In contrast, confidence was negatively associated with COVID-19 vaccine hesitancy, indicating individuals who lack confidence have hesitancy to receive COVID-19 vaccine. Confidence is defined as trust in the effectiveness and safety of vaccines, the system that delivers them, and the motivations of policy-makers who decide on the need of vaccines [3, 63]. During the COVID-19 pandemic, the rapid pace of vaccine development and ubiquitous misinformation could cause public’s concerns or distrust about the effectiveness and safety of COVID-19 vaccines, which undermined their confidence of the vaccine [64–66]. Distrusting the COVID-19 vaccine may stem from having little confidence in the governments or the health system since vaccination is usually a government-led public health intervention [67]. Moreover, lack of transparency in information about the development, approval, and use of vaccines may increase public distrust of the authorities involved. Therefore, ensuring and facilitating public access to reliable information from vaccine providers (e.g., health workers) and health authorities can facilitate the reduction of misinformation and increase public trust in authorities. In addition, health authorities should have the ability to utilize social media to monitor trends in public opinions and respond timely, thereby increasing the credibility of the government and relevant authorities in vaccination activities, thus improving or restoring public confidence in vaccines and reducing vaccine hesitancy.

As a specific instrument to assess COVID-19 vaccine hesitancy, the COVID-19 Vaccine Hesitancy Scale has advantages compared to other scales. The results of the current study indicated that the COVID-19 vaccine hesitancy scale could properly assess the population in a variety of backgrounds, including medical workers, students, public health professionals, and the general population. Additionally, this scale focused on three variables (“Behavior”, “Safety and Efficacy”, and “General Attitudes”) that are distinct from those of other scales and can be used to investigate multiple vaccine hesitancy reasons. Additionally, we concentrated on customizing the scale to the precise COVID-19 vaccine, which is more reliable to measure, in comparison to the previously validated general vaccine hesitancy scales. In the context of the ongoing global COVID-19 pandemic, there are still people who have not been vaccinated for various reasons, which undoubtedly hinders the global control of the COVID-19 pandemic. And, due to the emergence of variants, people who have already been vaccinated will need to receive a booster shot, a process that can also lead to vaccine hesitancy. The COVID-19 Vaccine Hesitancy Scale can measure COVID-19 vaccine hesitancy in this situation, which not only facilitates the identification of sources of hesitancy but also provides a reference for interventions to improve vaccination rates, which is crucial for lowering COVID-19 vaccine hesitancy.

### Limitations

Although the present study revised the COVID-19 vaccine hesitancy scale specifically for the COVID-19 vaccine and also validated the scale in different populations, limitations were still existed. First, all data were collected cross-sectionally using self-report, leading to the presence of information bias. Second, despite the large sample size containing a diverse population when considering the demographic data of the participants, a convenience sample was employed for the survey, which was not fully representative of the Chinese population. Besides, its psychometric properties have not been tested in populations from other countries because only Chinese people were recruited as study subjects in the present study. Further studies considering all these limitations are warranted to apply this scale among other different populations.

## Conclusions

The COVID-19 vaccine hesitancy scale was adapted and validated among different populations in China, and had good validity and reliability, which will assist in assessing vaccine hesitancy status and influencing factors, developing prevention or intervention programs, and improving vaccination rates. The validity of the COVID-19 vaccine hesitancy scale would need to be further evaluated across regions and time to increase its validity and generalizability.

## Supplementary Information

**Additional file 1:** COVID-19 Vaccine Hesitancy Scale Item Description.

## Data Availability

The datasets generated and/or analysed during the current study are not publicly available due to the confidentiality related to the protection of research participants, but are available from the corresponding author on reasonable request.

## Abbreviations

WHO: World Health Organization
COVID-19: Coronavirus disease 2019
VHS: Vaccine Hesitancy Scale
PACV: Parent Attitudes about Childhood Vaccines scale
CDC: Centers for Disease Control and Prevention
CVHS: COVID-19 vaccine hesitancy scale
EFA: Exploratory factor analysis
CFA: Confirmatory factor analysis
ICCs: Intraclass correlation coefficients
KMO: Kaiser–Meyer–Olkin
RMSEA: Root mean square error of approximation
CFI: Comparative fit index
TLI: Tucker Lewis index
AVE: Average variance extracted
CR: Composite reliability
